# Mitochondrial gene expression in different organs of *Hoplobatrachus rugulosus* from China and Thailand under low-temperature stress

**DOI:** 10.1186/s40850-022-00128-7

**Published:** 2022-05-19

**Authors:** Wan-Ting Jin, Jia-Yin Guan, Xin-Yi Dai, Gong-Ji Wu, Le-Ping Zhang, Kenneth B. Storey, Jia-Yong Zhang, Rong-Quan Zheng, Dan-Na Yu

**Affiliations:** 1 College of Chemistry and Life Science, Zhejiang Normal University, Jinhua, Zhejiang Province China; 2 Department of Biology, Carleton University, Ottawa, Canada; 3 Key Lab of Wildlife Biotechnology, Conservation and Utilization of Zhejiang Province, Zhejiang Normal University, Jinhua, Zhejiang Province China; 4 Xingzhi College, Zhejiang Normal University, Jinhua, China

**Keywords:** *Hoplobatrachus rugulosus*, Low-temperature stress, *RT*-qPCR, Mitochondrial gene expression

## Abstract

**Background:**

*Hoplobatrachus rugulosus* (Anura: Dicroglossidae) is distributed in China and Thailand and the former can survive substantially lower temperatures than the latter. The mitochondrial genomes of the two subspecies also differ: Chinese tiger frogs (CT frogs) display two identical *ND5* genes whereas Thai tiger frogs (TT frogs) have two different *ND5* genes. Metabolism of ectotherms is very sensitive to temperature change and different organs have different demands on energy metabolism at low temperatures. Therefore, we conducted studies to understand: (1) the differences in mitochondrial gene expression of tiger frogs from China (CT frogs) versus Thailand (TT frogs); (2) the differences in mitochondrial gene expression of tiger frogs (CT and TT frogs) under short term 24 h hypothermia exposure at 25 °C and 8 °C; (3) the differences in mitochondrial gene expression in three organs (brain, liver and kidney) of CT and TT frogs.

**Results:**

Utilizing *RT*-qPCR and comparing control groups at 25 °C with low temperature groups at 8 °C, we came to the following results. (1) At the same temperature, mitochondrial gene expression was significantly different in two subspecies. The transcript levels of two identical *ND5* of CT frogs were observed to decrease significantly at low temperatures (*P* < 0.05) whereas the two different copies of *ND5* in TT frogs were not. (2) Under low temperature stress, most of the genes in the brain, liver and kidney were down-regulated (except for *COI* and *ATP6* measured in brain and *COI* measured in liver of CT frogs). (3) For both CT and TT frogs, the changes in overall pattern of mitochondrial gene expression in different organs under low temperature and normal temperature was brain > liver > kidney.

**Conclusions:**

We mainly drew the following conclusions: (1) The differences in the structure and expression of the *ND5* gene between CT and TT frogs could result in the different tolerances to low temperature stress. (2) At low temperatures, the transcript levels of most of mitochondrial protein-encoding genes were down-regulated, which could have a significant effect in reducing metabolic rate and supporting long term survival at low temperatures. (3) The expression pattern of mitochondrial genes in different organs was related to mitochondrial activity and mtDNA replication in different organs.

## Background

Owing to a poikilotherm characteristic of being highly sensitive to temperature changes [1], when amphibians are subjected to low-temperature stress, they regulate their metabolism and initiate a series of cold-resistance mechanisms. In order to reduce damage to their bodies, the expression of cold resistance-related genes can be activated and regulated along with the synthesis of anti-stress-related substances [2, 3]. For instance, the transcription level of an antifreeze gene, *fr10*, in wood frogs (*Rana sylvatica*) can be regulated. FR10 and other related antifreeze proteins are synthesized to avoid damage to the organism under low-temperature stress [2]. Moreover, both *NFAT5* transcription factor and aldose reductases were up-regulated in the liver of wood frogs at low temperatures [4] and multiple other genes were also cold-responsive in frogs [5]. Increasingly, the importance of differential gene expression in the adaptation of amphibians to low temperature stress has been demonstrated [2–4].

Due to the inherently high levels of organizational complexity, gene transcripts of amphibians can demonstrate tissue-specific patterns in response to low-temperature conditions. For example, when wood frogs were subjected to low temperature freezing stress, miRNA-16 transcription level was significantly increased by a factor of 1.5 in the liver of frogs after freezing, but decreased by 50% in skeletal muscle [6]. Another study on wood frogs showed that tissue specific responses were observed during freezing, with phospho-Smad3 levels increased differently in brain, heart and liver [7]. For frogs, proper brain functions and adaptations to low temperature play a crucial role in coordinating stress responses [8]. In addition, liver is an important digestive gland and also the largest organ of amphibians, with the status of a metabolic center [9]. Kidney is also a very important organ that uses a lot of energy for ion regulation and excretion of water and waste products [10]. Hence, gene expression in these three organs (brain, liver and kidney) may have different responses and adaptations of metabolism to low temperature stress.

Although previous studies have focused mainly on changes in nuclear gene expression in amphibians under low temperature stress [5], the adaptation of mitochondrial gene expression changes to a low temperature environment has gradually received attention [11–14]. Mitochondria are the center of aerobic ATP production, housing both the citric acid cycle and the electron transport chain that ends in oxidative phosphorylation (OXPHOS) to produce ATP [15]. Mitochondria can play significant roles in the adaptation of organisms to environmental temperature changes [16]. A growing body of research suggest that temperature change can lead to changes in the expression of protein-coding genes in different mitochondrial complexes [13, 17–22], which could be a considerable benefit for reorganizing metabolism to adapt to changing environmental temperature [17]. Indeed, transcripts of the mitochondrial genes *ATP6/8*, *ND4* and *16 S RNA* in wood frogs were strongly up-regulated in the liver and brain during whole body freezing (24 h at –2.5 °C) [14]. By contrast, in a study of the response of *Dryophytes versicolor* mitochondrial protein-coding gene under freezing stress, it was observed that the relative transcription levels of the *COI* gene decreased [13]. In cold storage conditions, a down-regulation of gene clusters composed of *ND4*, *ND5*, *ND6*, and *COIII* genes was also found in embryos of *Coturnix chinensis*, which suggested a slow-down of metabolic rate [23]. Low temperature inhibits metabolic rate, reducing the need for ATP and the consumption of endogenous fuel reserves, and thus can prolong animal survival in harsh environments [24–31].

The tiger frog (*Hoplobatrachus rugulosus*) belongs to the anuran family Dicroglossidae. There are two distinct populations of tiger frogs in China. One is imported from Thailand and shows a fast growth rate and high-temperature resistance, and is called the Thai tiger frog (TT frog). The other is native to China, has a slower growth rate and greater low-temperature resistance, and is called the Chinese tiger frog (CT frog). CT frogs can endure temperatures as low as –2 °C, whereas TT frogs die below 4 °C. Both are edible frogs and are widely farmed in southeast Asia and sold in markets. CT frogs induce immunosuppression and increase the demand for antioxidant substances at low temperatures, resulting in a slower growth rate than that of TT frogs [32]. Due to this slower growth rate, not many farms are willing to raise CT frogs, but many farmers have to heat their farm’s facilities in the winter to prevent TT frogs from dying due to cold or freezing. Furthermore, due to low ambient temperatures in winter, TT frogs cannot be bred in vast areas of northern Zhejiang, China. Although there is little difference in morphological characteristics between CT and TT frogs, the genetic distance in the mitochondrial genome can reach up to 14% between them [33, 34]. In particular, two identical *ND5* genes were found in CT frogs [33], whereas two different *ND5* genes (84.1% similar sequence) were found in TT frogs [34]. These facts led us to wonder whether CT and TT frogs have differences in mitochondrial gene expression under low-temperature stress, which may provide an opportunity for a new research direction to breed a new strain of tiger frogs that exhibit both fast growth rate and low temperature resistance.

In this study, we analyzed the differential mitochondrial gene expression of the 13 mitochondrial protein-coding genes (PCGs) in brain, liver and kidney of CT and TT frogs, comparing frogs held at 25 °C as the control group with frogs at 8 °C as the low-temperature group. The research aimed to answer three questions: (1) Is there a difference in mitochondrial gene expression of the 13 PCGs, especially the *ND5* gene, between CT and TT frogs? (2) Do CT and TT frogs exhibit different mitochondrial gene expression profiles under low-temperature stress? (3) Are there differences in mitochondrial gene expression in different organs of CT and TT frogs?

## Results

### Differential gene expression in chinese and thai tiger frog under the same temperature conditions

**Fig. 1:**
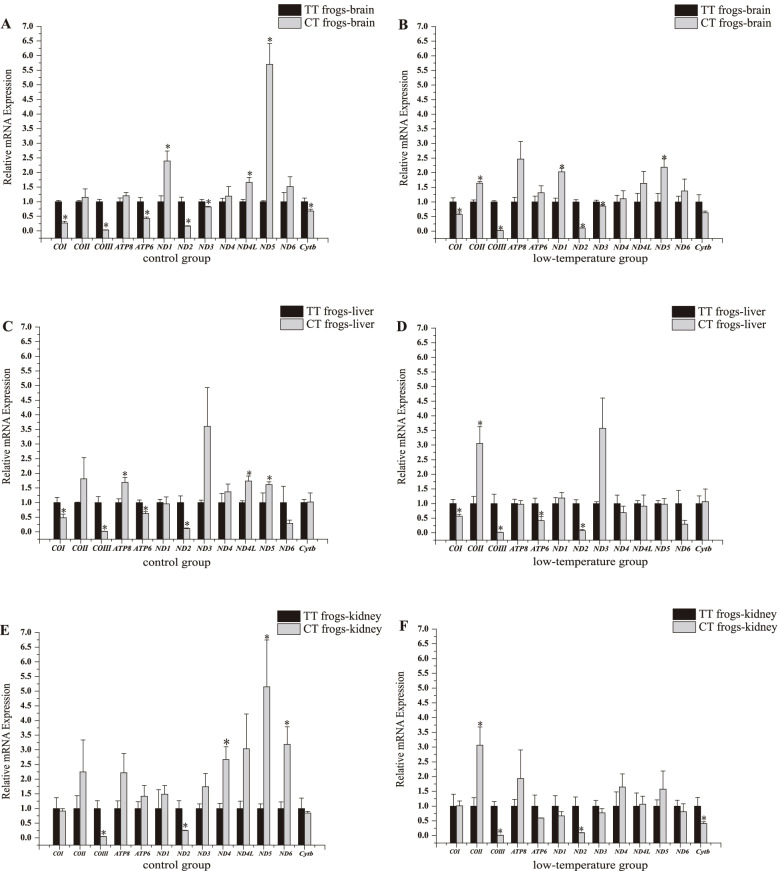
Relative mRNA expression levels of protein-coding mitochondrial genes in brain, liver and kidney of normal temperature 25 °C (**A**, **C**, **E**) and low temperature 8 °C (**B**, **D**, **F**) exposed *H. rugulosus* (TT frogs) and *H. rugulosus* (CT frogs). Relative mRNA levels were determined by *RT*-qPCR with *n* = 4 independent biological replicates and results are shown as mean ± SE. Mean values for TT frogs were set to 1.0 and values for CT frogs are expressed relative to TT frogs. Relative transcript levels were standardized against *β-actin* transcript levels as the reference gene. For each sample, the average *β-actin* Ct value was subtracted from the average target Ct value, obtaining a ΔCt value. Data are shown as relative expression, calculated as 2^-ΔΔCt^. Statistical significance was assessed with a two-tailed Student’s t-test, asterisks indicate significantly different expression (*, *P* < 0.05) and (**, *P* < 0.01)

We identified differential gene expression in the three organs tested from CT and TT frogs held under identical temperature conditions. Mean values for gene expression in TT frogs were standardized to 1.0 ± SEM and values for CT frogs were expressed relative to TT frogs. For mitochondrial gene expression in the brain, some genes showed higher expression in CT frogs compared with TT frogs (Fig. 1A-B). In the brain of frogs held at 25 °C, we found that mitochondrial gene transcript levels of *ND1*, *ND4L* and *ND5* among CT frogs were 2.39 ± 0.34, 1.66 ± 0.17, and 5.70 ± 0.71 times higher, respectively, as compared to values in TT frogs (*P* < 0.05) (Fig. 1A). Transcripts of *ND1* and *ND5* in the brain of CT frogs after 8 °C low-temperature stress were also higher by 2.03 ± 0.12 and 2.19 ± 0.29 fold compared with those in TT frogs, and *COII* transcripts were also elevated by 1.64 ± 0.06 fold in the cold (Fig. 1B). Oppositely, transcript levels of some genes were reduced in CT frogs compared with TT frogs. Under 25 °C normal conditions, the mitochondrial gene expression of *COI*, *COIII, ATP6, ND2, ND3*, *ND3*, and *Cytb* genes in the brain of CT frogs were significantly reduced to values of 0.27 ± 0.06, 0.03 ± 0.01, 0.43 ± 0.04, 0.16 ± 0.01, 0.82 ± 0.02, and 0.68 ± 0.06, respectively, as compared with those in brain of TT frogs (*P* < 0.05) (Fig. 1A). Analysis of the low-temperature exposure groups also showed significant differences in mitochondrial gene transcript levels of *COI*, *COIII, ND2* and *ND3* which were just 0.57 ± 0.05, 0.03 ± 0.003, 0.11 ± 0.003, and 0.86 ± 0.03, respectively, as compared with those in brains of TT frogs (*P* < 0.05) (Fig. 1B).

Liver also showed significant changes in mitochondrial gene expression both between species and in response to cold exposure (Fig. 1C-D). When comparing mitochondrial gene expression at normal temperature (25 °C) in the liver, we found that transcript levels of *ATP8, ND4L* and *ND5* genes in CT frogs were 1.69 ± 0.17, 1.74 ± 0.17 and 1.62 ± 0.09 times higher, respectively, than in TT frogs (*P* < 0.05) (Fig. 1C). However, only *COII* transcript levels were increased significantly by 3.06 ± 0.57 fold in CT frogs held at low 8 °C temperature, as compared with TT frogs (*P* < 0.05) (Fig. 1D). By contrast, *COI*, *COIII, ATP6*, and *ND2* gene transcripts were significantly reduced in liver of CT frogs under both normal and low temperatures. Transcript levels of *COI*, *COIII, ATP6*, *ND2* in CT frogs were just 0.48 ± 0.12, 0.02 ± 0.00, 0.63 ± 0.07, and 0.11 ± 0.02, respectively, relative to TT frogs held at 25 °C, and were 0.57 ± 0.06, 0.01 ± 0.01, 0.42 ± 0.14, 0.07 ± 0.04 relative to TT frogs at 8 °C, respectively (Fig. 1C-D). Hence, under both 25 °C and 8 °C conditions, *COI*, *COIII, ATP6* and *ND2* showed the same pattern with transcript levels of all four significantly lower in CT frogs compared to TT frogs (*P* < 0.05).

In the case of kidney, transcript level of *ND4*, *ND5, ND6* genes in kidney of CT frogs under normal conditions were 2.67 ± 0.44, 5.15 ± 1.59, 3.19 ± 0.6 times higher than those in TT frogs, respectively, (*P* < 0.05) (Fig. 1E). Under low temperature exposure, however, relative transcript levels changed substantially with only *COII* transcripts being significantly higher in CT frogs (by 3.06 ± 0.62 fold) as compared with TT frogs (*P* < 0.05) (Fig. 1F). By contrast, the relative expression of *COIII* and *ND2* genes in the kidney of CT frogs was significantly lower under normal conditions than in TT frogs, with values of 0.05 ± 0.00 and 0.25 ± 0.01 compared with TT frogs (*P* < 0.05). At 8 °C, kidney mitochondrial gene transcript levels of *COIII* and *ND2* in CT frogs showed the same pattern as at 22 °C with levels of 0.01 ± 0.00 and 0.09 ± 0.01 compared with TT frogs. In addition, *Cytb* transcripts were also reduced in kidney of CT frogs after low-temperature exposure 0.41 ± 0.06 relative to TT frogs (*P* < 0.05).

**Fig. 2:**
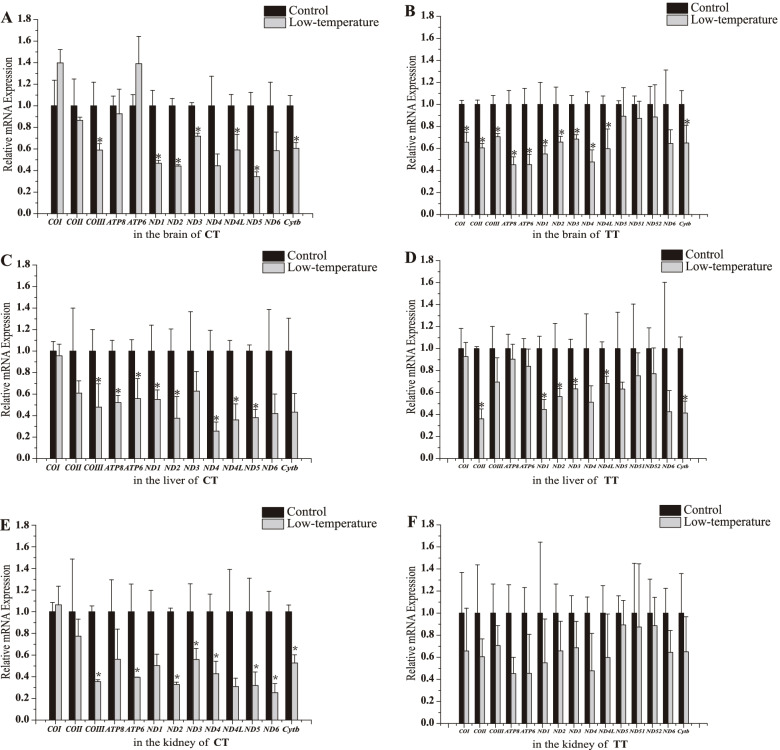
Relative mRNA expression levels of protein-coding mitochondrial genes in brain, liver and kidney before and after low temperature 8 °C stress in *H. rugulosus* (CT frogs) (**A**, **C**, **E**) and *H. rugulosus* (TT frogs) (**B**, **D**, **F**). Relative mRNA levels were determined with *RT*-qPCR with *n* = 4 independent biological replicates and results are shown as mean ± SE. Mean values for controls were set to 1.0 and values for low-temperature groups are expressed relative to controls. Relative transcript levels were standardized against *β-actin* transcript levels as a reference gene. For each sample, the average *β-actin* Ct value was subtracted from the average target Ct value, obtaining a ΔCt value. Expression is represented as relative expression, calculated as 2^-ΔΔCt^. Statistical significance was assessed with a two-tailed Student’s *t*-test, asterisks indicate significantly different expression (*, *P* < 0.05) and (**, *P* < 0.01)

It is remarkable that in liver, kidney and brain of CT frogs, the *ND5* gene with two identical copies showed significant differences under low temperature stress. In addition, what surprised us was a highly significant difference found in liver. In the three organs of TT frogs, the transcript levels of the two different copies of *ND5* genes were both detected, and the total *ND5* gene expression in TT frogs was approximately the sum of two different *ND5* gene expressions. By comparison, there was no significant differences in the transcript level of two different *ND5* genes and the total *ND5* gene after low-temperature stress in the three organs of TT frogs (Fig. 2). Given the above, for TT and CT frogs, only the *ND5* gene expression difference is peculiar to CT frogs after low-temperature stress in the three organs.

### Effects of low-temperature stress on gene expression of two types of tiger frog

Figure 2 show the relative changes in mRNA transcript expression of mitochondrial genes after cold exposure of CT and TT frogs. After 24 h of low temperature exposure at 8 °C, transcript levels of the 13 mitochondrial protein-coding genes (PCGs) of CT and TT frogs in all three organs examined (brain, liver, kidney) were either significantly reduced or unchanged compared with values in frogs that held at 25 °C (Fig. 2). However, *COI* and *ATP6* measured in brain of CT frogs and *COI* measured in kidney of TT frogs showed an upward trend in cold-exposed frogs, but the values were not significantly different from the controls (Fig. 2). Oppositely, PCG transcript levels in kidney of TT frogs all showed downward trends compared with the controls but also very large SEM values such that no significant differences occurred.

**Fig. 3:**
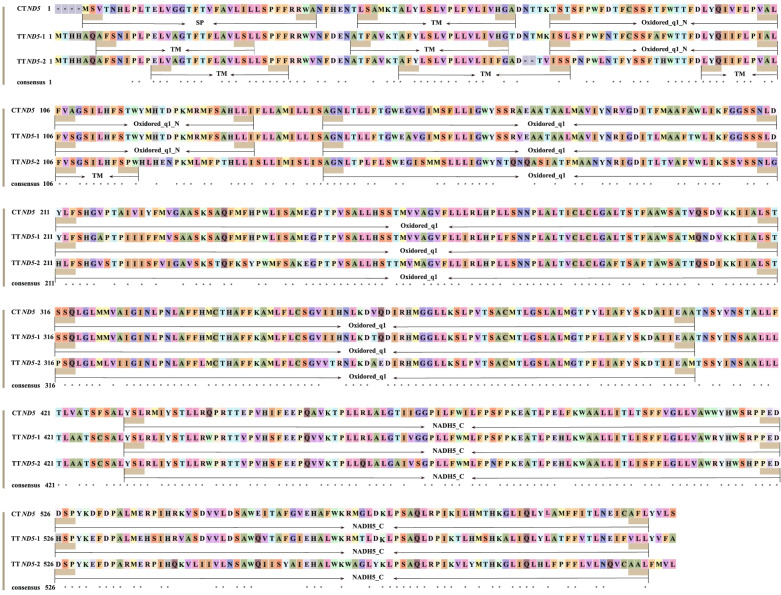
Alignment of the amino acid sequences and their structures of domain of *ND5* genes in CT frogs and *ND5-1* and *ND5-2* genes in TT frogs. Different shaded colors represent different amino acid. The symbol “*” indicates that at this site, the amino acid of *ND5* gene of CT frogs is the same as that of *ND5-1* and *ND5-2* genes of TT frogs

In the brain of CT frogs, after low-temperature treatment at 8 °C, the transcript levels of seven PCGs decreased significantly with relative decreases in transcript levels of *COIII*, *ND1*, *ND2*, *ND3*, *ND4L*, *ND5* and *Cytb* genes to 0.59 ± 0.06, 0.47 ± 0.03, 0.44 ± 0.01, 0.72 ± 0.03, 0.59 ± 0.15, 0.34 ± 0.05, 0.61 ± 0.05, respectively, as compared with controls (*P* < 0.05) (Fig. 2A). In addition, *COI* and *ATP6* gene expression showed an upward trend but this was not significant. By comparison, in brain of TT frogs, 11 out of 13 PCGs’ transcript levels and the total and respective protein coding gene transcript level of *ND5-1* and *ND5-2* showed a significant decrease at low temperature. Transcript levels of *COI*, *COII COIII*, *ATP8*, *ATP6*, *ND1*, *ND2*, *ND3*, *ND4*, *ND4L* and *Cytb* genes were reduced to 0.66 ± 0.09, 0.60 ± 0.04, 0.71 ± 0.03, 0.45 ± 0.07, 0.45 ± 0.09, 0.55 ± 0.07, 0.66 ± 0.05, 0.69 ± 0.04, 0.48 ± 0.11, 0.60 ± 0.18 and 0.65 ± 0.16, respectively, under low temperature exposure as compared to controls (*P* < 0.05) (Fig. 2B). The common ground between TT and CT frogs responses in brain was the significant decreases in transcript levels of *COIII*, *ND1*, *ND2*, *ND3*, *ND4L* and *Cytb* (*P* < 0.05) in the cold. However, *COI*, *COII*, *ATP8*, *ATP6* and *ND4* were down-regulated significantly only in TT frogs, whereas *ND5* was down-regulated significantly only in CT frogs (*P* < 0.05) (Fig. 2A-B). As mentioned earlier, the mitogenome of TT frogs contains two *ND5* additional, but two different *ND5* genes (84.1% similar sequence) (Fig. 3) were found in TT frogs [34]. The total and respective expression of two different *ND5* genes (*ND5-1* and *ND5-2*) in TT frogs were unaffected by temperature change (Fig. 2).

In the liver of CT frogs, transcript levels of 8 of the 13 PCGs decreased in response to 24 h low temperature exposure. Significant differences in the transcript levels of *COIII, ATP8, ATP6, ND1, ND2, ND4, ND4L*, and *ND5* genes lead to levels of 0.48 ± 0.22, 0.52 ± 0.07, 0.56 ± 0.19, 0.55 ± 0.09, 0.38 ± 0.20, 0.26 ± 0.08, 0.36 ± 0.15 and 0.38 ± 0.08, respectively, as compared to respective controls (*P* < 0.05) (Fig. 2C). However, in liver of TT frogs, fewer PCGs showed decreased expression under low-temperature stress; transcript levels of *COII, ND1*, *ND2*, *ND3, ND4L* and *Cytb* genes were reduced to 0.36 ± 0.09, 0.45 ± 0.09, 0.56 ± 0.07, 0.63 ± 0.04, 0.68 ± 0.07 and 0.41 ± 0.11, respectively, as compared to the controls (*P* < 0.05) (Fig. 2D). Gene transcript levels of *ND1*, *ND2* and *ND4L* in liver were significantly reduced in both CT and TT frogs in response to cold.

As for kidney, the transcript levels of *COIII*, *ATP6*, *ND2*, *ND3*, *ND4*, *ND5*, *ND6*, and *Cytb* genes in CT frogs decreased strongly in response to 8 °C exposure with values only 0.35 ± 0.02, 0.40 ± 0.00, 0.33 ± 0.02, 0.56 ± 0.10, 0.43 ± 0.12, 0.32 ± 0.12, 0.25 ± 0.08 and 0.53 ± 0.07, respectively, as compared to the control group (*P <* 0.05) (Fig. 2E). However, among TT frogs, unlike liver and brain, expression of genes tended to be downward, but there were no significant differences between control and low-temperature treatments, largely due to high variance among individuals (Fig. 2F).

### Effects of low-temperature stress on gene expression in different organs

**Fig. 4:**
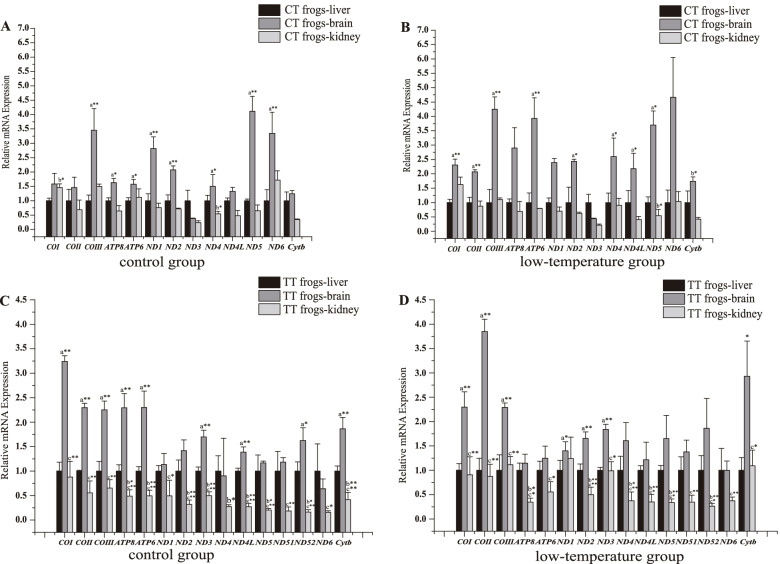
Relative mRNA expression levels of protein-coding mitochondrial genes in kidney, brain, and liver of *H. rugulosus* (CT frogs) and *H. rugulosus* (TT frogs) under normal conditions (24 h at 25 °C) (**A**, **C**) and low-temperature exposure (24 h at 8 °C) (**B**, **D**) Mitochondrial gene expression levels in each tissue were standardized relative to *β-actin* transcript levels. Statistical significance was assessed with a two-tailed Student’s t-test, “a” indicates significantly different expression between brain and liver (a*, *P* < 0.05) and (a**, *P* < 0.01); “b” indicates significantly different expression between liver and kidney (b*, *P* < 0.05) and (b**, *P* < 0.01); and “c” indicates significantly different expression between brain and kidney (c*, *P* < 0.05) and (c**, *P* < 0.01)

At 25 °C, mitochondrial gene expression of brain genes in CT samples were significantly higher overall than in kidney (except for *COI*, *COII*, *ATP6* and *ND4*) and also higher than in liver (except for *COII*, *ND3*, *ND4L* and *Cytb*) (Fig. 4A). Furthermore, except for *COI*, *COIII*, *ATP6* and *ND6* genes, levels of other mt gene transcripts were also higher in liver of CT frogs than in kidney at 25 °C and *ND4* gene reached a significant level (*P* < 0.05) (Fig. 4A). When comparing mitochondrial gene transcript levels of the three organs under low-temperature stress (8 °C), the transcript levels in brain of all CT frogs also showed significantly higher levels (*P* < 0.05) than in kidney except for *ND6*, and mitochondrial gene expression was also higher in brain than in liver (except for *ND3* gene, which was higher in liver and *ATP8*, *ND1*, *ND6*, *Cytb* that did not reach a significant level) (Fig. 4B). Under low-temperature stress, except for *COI, COIII*, *ATP6* and *ND6* genes, expression of other mitochondrial genes in the liver of CT frogs was higher than those in the kidney but only *ND5* and *Cytb* reached a significant level (Fig. 4B).

Similar results were found for TT frogs. At 25 °C, mitochondrial gene transcript levels in brain of TT frogs were significantly higher than those in kidney for all genes (*P* < 0.05), except *ND4*. Transcript levels in brain were also significantly higher than 9 genes in liver, but not for *ND1, ND2, ND4, ND5, ND5-1* and *ND6* (Fig. 4C). Furthermore, mitochondrial transcript levels of liver genes in all TT frogs were significantly higher than those in the kidney at 25 °C (*P* < 0.05), except for the 3 *COX* genes as well as *ND1*, *ND5-1* and *ND6* genes (Fig. 4C). When comparing the gene expression of the three organs in frogs exposed to 8 °C for 24 h, it was clear that mitochondrial gene expression in brain after low-temperature stress was also significantly higher than in kidney (except for *ND1*). Expression in brain was also higher than most genes in liver (except for *ATP8*, *ATP6*, *ND4*, *ND4L*, *ND5* and *ND6* not reached a significant level) (Fig. 4D). Under low-temperature stress, except for *COIII, ND1* and *Cytb* genes, mitochondrial gene transcript level of other genes in the liver of TT frogs were higher than those in the kidney and *ATP8*, *ND2*, *ND4*, *ND4L* and *ND5-2* reached a significant level (*P* < 0.05) (Fig. 4D). In conclusion, under normal and low temperature, the expression of genes in three organs of TT and CT frogs was roughly as follows: brain > liver > kidney.

## Discussion

### Different mt gene expression between chinese and thai tiger frog

The tolerance of CT frogs to low temperatures is greater from that of TT frogs. CT frogs can tolerate temperatures as low as − 2 °C, whereas the TT frogs will die below 4 °C. The differences observed between CT and TT frogs in the expression of mitochondrial protein-coding genes under either normal or low-temperature conditions were generally unambiguous. Significant differences in gene transcript levels within the same species living in different regions suggest adaptation to prevailing temperature ranges occurring in each region. Similar responses have been reported for other ectothermic species. For example, cold (4 °C) and warm (10 °C) acclimated *Gadus morhua* from cod populations of the North Sea and the Barents Sea show a strong difference in gene expression at 4 °C, which may reflect the stable separation of both populations [35]. Differential transcriptional responses of common carp (*Cyprinus carpio*) subjected to a progressive cooling regime also suggested that ectothermic animals might experience complex transcriptional changes in the process of temperature adaptation, and that the low growth rate of populations adapted to low temperatures is one of the costs of low temperature adaptation [36]. Indeed, the smaller body sizes of CT frogs may be due to their lower metabolic rate imposed by lower ambient temperatures. It can be a long evolutionary process for amphibians to adapt to a lower temperature environment. When an organism is subjected to low-temperature stress, it will regulate its metabolism and initiate a series of cold-resistant mechanisms [2]. One way that amphibians can adapt to changing environmental temperatures is by regulating the transcript levels of mitochondrial protein-coding genes and synthesizing other stress-resistance related substances [14, 37]. The temperature-adaptive transformation of gene expression is the basis of physiological adaptation [38]. In our study, significant differences in mitochondrial gene expression between frogs from two regions reflected a mitochondrial bioenergy adaptation to low temperature. However, the specific significance of these changes in gene transcript levels in the process of low temperature adaptation remains unclear.

In the two populations of tiger frogs, two identical copies of the *ND5* gene were found in CT frogs [33], whereas two additional and different *ND5* genes (*ND5-1, ND5-2*) were found in TT frogs (Fig. 3) [34]. The *ND5* gene with two identical copies in CT frogs showed significant differences in expression under low-temperature stress, especially in the liver, whereas there were no significant differences in the transcript levels of the two different *ND5* genes and the total *ND5* gene content in TT frogs (Fig. 2). The *ND5* gene expression difference in the three organs was peculiar to CT frogs after low-temperature stress. The ability to regulate gene expression requires open DNA structures that appear to be closely regulated in homeostasis and cyclic heat states. Being able to regulate the transcriptional capacity of mitochondrial DNA in the face of changing temperatures in order to ensure the necessary changes in gene expression may be a basic requirement for temperature adaptation [38]. Hence, a different DNA structure of the *ND5* gene may also be an important reason for the difference in low temperature tolerance between CT and TT frogs. Indeed, mutations in the *ND5* gene are considered to be a common cause of oxidative phosphorylation disease [39]. Studies have shown that mutations in *ND5* may cause changes in the activity of NADH dehydrogenase (complex I of the respiratory chain) and affect other enzymes of the respiratory chain [40]. In addition, *ND5* gene mutation may lead to a decrease in mitochondrial complex I activity [41], and is suggested to be associated with assembly defects of the whole respiratory system complex [42]. Thus, mutations in *ND5* may lead to decreases in membrane potential difference and result in reduced ATP production [43, 44]. 288 variable sites in a total of 1848 aligned nucleotide sites and 131 variable sites in amino acid sequence over a total of 616 alignment amino acid sites were found comparing the two *ND5* genes in TT frogs (Fig. 3). According to three-dimensional structure analysis of the *ND5* proteins, the two *ND5* genes in CT frogs both contain two *β*-sheets and 22 α-helices, and their structures are highly similar. Correspondingly, *ND5-1* in TT frogs contains two *β*-layers and 22 α-helices, and *ND5-2* contains two *β*-layers and 23 α-helices [34]. Studies showed that the membrane protein completed a single or multiple transmembrane by α-helix [45]. The mitochondrial *ND5* subunit of TT frogs had more α-helix than in CT frogs, which enlarges the region of transmembrane protein and could enhance the coupling degree of the mitochondrial *ND5* subunit to a certain extent in the oxidative phosphorylation of TT frogs. Combined with the differences in *ND5* expression and gene structure, *ND5* gene transcript levels in CT frogs decreased significantly in tissues including liver, brain and kidney under low-temperature stress, potentially leading to less coupling of oxidative phosphorylation (i.e. more proton leakage) and resulting in less harmful reactive oxygen species and more heat, which could help to combat a cold environment [46–48].

### Low-temperature stress on mt gene expression

In this study, we sought to determine if environmental temperature might influence the transcript levels of mitochondrial protein-coding genes in three organs (brain, liver and kidney) of CT and TT frogs. Our results showed that low-temperature stress decreased the transcript levels of mitochondrial protein-coding genes in all three tissues of tiger frogs examined (Fig. 2). In brain, the significant decreases in transcript levels of *COIII*, *ND1*, *ND2*, *ND3*, *ND4L* and *Cytb* (*P* < 0.05) were found in the cold of CT and TT frogs. Gene transcript levels of *ND1*, *ND2* and *ND4L* in liver were significantly reduced in both CT and TT frogs in response to cold. In kidney, most genes showed significant downregulation in CT frogs, but in TT frogs, only downward trend was detected, but no significant difference was found. Several previous studies have also demonstrated similar relationships between mitochondria and low-temperature exposure. For example, Colinet et al. [49] found that both mitochondrial respiration and ATP synthesis rate of *Drosophila melanogaster* would temporarily decrease under exposure to low temperature. Cold exposure also led to an extreme reduction of the resting metabolic rate of *Bufo marinus*, which showed an adaptation of mitochondrial bioenergetics [50]. Similar results were also observed at the gene expression level, *COI* transcript level was decreased with exposure to freezing in *D. versicolor*, as part of the modulation of mitochondrial protein-coding gene expression in the molecular response to freezing [13]. Mitochondrial respiration rate is closely related to the metabolic regulation of cells [51]. Oxidative phosphorylation is the metabolic pathway in which ATP is produced through a series of biochemical reactions in mitochondria. It has been shown that ectotherms inhibit their metabolic rate, reducing the impact of ATP demand on endogenous fuel reserves, and thus extending their survival time in harsh environments, including cold climates [28–31, 52]. For ectotherms like tiger frogs, that are sensitive to temperature [53], low temperature stress resulted in down-regulation of mitochondrial gene expression and inhibition of metabolic rate, which prolonged survival at low temperatures.

In addition, under low temperature stress, the number of down-regulated genes (11 genes) in brain of TT frogs was higher than that in CT frogs (7 genes), whereas in kidney, eight genes were significantly down-regulated in CT frogs (*P* < 0.05), but no significantly down-regulated genes were found in TT frogs. Studies have suggested that gene expression in the brain is more complex than in other parts of an organism to reflect the diverse functions of neurons and glia [54]. In addition, neurons showed other forms of “activations” in brains of frozen wood frogs including changes in protein kinase C phosphorylation status, and changes in acidic ribosomal phosphoprotein P0 and the large ribosomal subunit protein 7 [55–57]. Normal brain function and adaptation to environmental stimuli play crucial roles in coordinating the stress response [8]. Under low temperature stress, transcript levels of a large number of mitochondrial genes were significantly down regulated in the brain of TT frogs, which may be one of the important reasons for its intolerance to low temperature. The kidney is an important osmotic regulating organ of great significance for maintaining ion balance in organisms [58]. In addition, studies have shown that under low temperature stress, kidneys may not be able to excrete salt absorbed through frog skin, leading to an accumulation of water and salt in their bodies [59]. Therefore, we speculated that in a low temperature environment, compared with TT frogs, the CT frogs show greater downregulation of mitochondrial gene expression in the kidney, which can reduce metabolic rate faster and increase its tolerance to low temperatures on the premise of reducing the damage to the body.

### Tissue heterogeneity in mt gene expression

Under low-temperature stress, expression of almost all genes were reduced compared to normal temperatures in brain, kidney and liver of both CT and TT frogs. However, there were significant differences among the gene transcript levels of the three different tissues, the changes in gene expression of the three organs of TT and CT frogs being roughly as follows: brain > liver > kidney. Mitochondria from different cell types are functionally unique and diverse in appearance [60, 61], likely to suit the needs of different cells and tissues that perform different metabolic functions and have different energy requirements. In general, in the present study, gene expression rates were higher in the brain than in liver and kidney. Studies have shown that mitochondrial activity and mitochondrial mass in brain are significantly higher than those in liver and kidney [62], which may lead to higher mtDNA copy numbers in the brain. That is consistent with our results that showed that mitochondrial gene expression in the brain was higher than that in the kidney and liver. In addition, Veltri et al. [63] analyzed the stereology of thin tissue sections in mice and found that the mitochondrial density in the kidney was slightly higher than that in the liver. However, a determination of mtDNA quantification revealed that the relative mtDNA content in liver was higher than that in kidney [63], which was consistent with our findings of mitochondrial gene expression that was roughly higher in liver than kidney. This may indicate that differences in mtDNA replication are indicators of the metabolic function of an organ, an idea supported by Herbers et al. [64]

## Conclusions

In our study, we found that tiger frogs from Chinese (CT frogs) versus Thai (TT frogs) populations showed significant differences in mitochondrial gene expression at different temperatures (25 °C versus 8 °C), which reflects prolonged adaptation of mitochondrial bioenergetics in these two subspecies to the prevailing environmental temperature conditions. Under low temperature stress, especially in the liver, the *ND5* gene of CT frogs with two identical copies showed significant differences, whereas in TT frogs, the expression of two different *ND5* genes and the total *ND5* gene expression did not differ significantly. The differences in the structure and expression of the *ND5* gene between CT and TT frogs could result in the different tolerances to low temperature stress of CT and TT frogs. In addition, under low temperature conditions, we found that the majority of mitochondrial genes showed down-regulation in the three organs. This reduction in mitochondrial protein-encoding gene transcript levels in response to lower temperatures could have a significant effect in reducing metabolic rate and supporting long term survival at low temperatures. In addition, compared with TT frogs, CT frogs showed more gene down-regulation in the kidney at low temperature, but gene down-regulation in the brain was the opposite. This may be because CT frogs can minimize metabolism while maintaining the important function of the brain. This may be one of the important reasons why CT frogs are more resistant to low temperatures than TT frogs. Furthermore, when comparing the expression differences between the three organs, we found that their expression showed the following general pattern: brain > liver > kidney, which is related to mitochondrial activity and mtDNA replication in different organs.

## Materials and methods

### Animal sources and cold stress treatment

*H. rugulosus* including CT and TT frogs were collected from a farm in Jinhua, Zhejiang province, China on 20 June 2018. All tiger frogs are adult females for one year with similar size. All animals were washed in a tetracycline bath and were fed in a plastic incubator at 25 °C for one week. Four CT frogs and four TT frogs from the 25 °C temperature groups were randomly selected and placed in a plastic box under a wet towel for 24 h at 25 °C as the control group. At the same time, four CT and TT frogs were exposed to low-temperature stress for 24 h at 8 °C as the low-temperature stress group. Subsequently, tiger frogs from both control and low-temperature groups were euthanized by pithing followed by rapid dissection of liver, brain and kidney and freezing in liquid nitrogen. Thereafter, organ samples were stored in an ultra-low temperature freezer at − 80 °C until use.

### RNA extraction and cDNA synthesis

Frozen samples (50–100 mg) of brain, liver and kidney from CT and TT frogs were used for RNA extraction using a TaKaRa MiniBEST Universal RNA Extraction Kit (Takara, Japan), according to manufacturer’s instructions. Samples were then subjected to electrophoresis on a 1% agarose gel and run at 135 V and 120 mA for 15 min followed by staining with Goldview. RNA integrity was verified by the presence of sharp bands for *28 S* and *18 S* ribosomal RNA [65]. RNA was then stored at -80 °C until use. Following the instructions of a PrimeScript™ RT Master Mix kit (Takara, Japan), sample volumes containing 500 ng of RNA were gently mixed for reverse transcription. Reaction conditions were as below: 37 °C, reverse transcription for 15 min; inactivation of the reverse transcriptase at 85 °C for 5 s.

### *RT*-qPCR primer design

Specific primers for reverse transcription-quantitative polymerase chain reaction (*RT*-qPCR) were designed according to the gene sequences of *H. rugulosus* [33, 34] downloaded from the NCBI website and using MegAlign (DNASTAR) and Primer Premier 5.0 software [66]. Primers were synthesized by related biotechnology companies and are listed in Table 1. The length of amplicons varied from 120 to 150 bp, melting temperatures were designed between 48 °C and 52 °C and the primer lengths were between 18 and 22 bp.

**Table 1:** *RT*-qPCR primers of *H. rugulosus* (TT frogs) and *H. rugulosus* (CT frogs)

Gene	Forward primers (5′–3′)	Reverse primers (5′–3′)
*TT-COI*	CGGCTTCGGAATCATCTC	TTAGGTCGGTCGTGAATATG
*CT-COI*	CTCCTTGGTGGGGGTTAT	GGCTTCTTCAAATGTGTGG
*TT-COII1(TT- COII2)*	CAACCGCATAGTAACT(C)CC	GGTGAGCAGTAATAAAAGCAG
*CT-COII*	CTGCTTTTATTACTGCCCA	TTAGTCAGGAGAGGAAGTTG
*TT-COIII*	GACGAGATGTTGTCCGAG	ATTGTAGAATGCCCAGAAG
*CT-COIII*	CTCCACAGTCCTTGTATTTG	AGAGGATTATGCCAAAGC
*TT-ATP8*	TGGTACATGGTCCTTCTCT	GTTCAGTGTCAGATGTTAGTC
*CT-ATP81(CT-ATP82)*	CCCTGGTACTCG(C)ATTCTTC	CGCTGCTTTGAGAAGTG(A)G
*TT-ATP6*	CTGTCATCCTTAGTGGTCTT	ATGAGAACGGTGGCTAAC
*CT-ATP61(CT-ATP62)*	ACTTTC(T)TACCAGAGGGCAC	AGGTGGATAAGCAGGTGAC
*TT-ND1*	GCTCTAATGTGATTCGTATCC	TTCGGCAAGGAAGAATAGG
*CT-ND1*	CAACTCCAAATACGCTCTC	TGGGCGTAGGTGAAGTTA
*TT-ND2*	TGACTACTCGCTTGAATCG	GTTGCTGAGAATAGGATAAGTG
*CT-ND2*	ATTCTCAGCGACCATAAAC	TTACTTCGGGCATTCAGA
*TT-ND3*	GTGCTCATATTCCTATCAGTC	CCGCATTCGTATGGAGATA
*CT-ND3*	CTACCCTTCTGGTCATCG	CAGGAAAAATCGTATTGAGTA
*TT-ND4*	AACAATCATCACCGTCCAA	GTCAGCAGGTTAGCAGAAT
*CT-ND41(CT-ND42)*	AAGGCTTCTTTATT(C)GCTGA	GGAA(G)TAGCCAGCAGGTTA
*TT-ND4L*	GGCATGATACTTACCATCTTC	GCTGTGGCAATCATTAAGG
*CT-ND4L1(CT-ND4L2)*	TCAACTGAAC(T)TCAACCACTC	AGAAGATTTAGCGTTTTGAG
*TT-ND5*	TACCTGTCCACTCCTTCG	TCTGGTAGTGTCGCTTCT
*CT-ND5*	TTTACAGTGTTTGCGGTC	ATAAGGACGAACAGTGGG
*TT-ND5-1*	AAGACACCCAAGACATTCG	TCCTTAGAGTAGAATGCGATG
*TT-ND5-2*	CCTCCTCCTCACTCTTGC	GTTTTTACGACTTGTGGTTC
*TT-ND6*	AATATAAGCCGCTAGGTAGAC	GGGTATAATGGTGGTATTTGC
*CT-ND6*	CCAACCCGTCAAACAACA	TTGCTTATAGTGCTGCTCTT
*TT-Cytb*	GGCGTTGTCCTTCTCTTC	AGGTTGGTGATGACTGTTG
*CT-Cytb*	GCTTTCGTGGGCTATGTC	AGTTAGCGTGGCGTTGTC
*Actin*	GATCTGGCATCACACTTTCT	TGGGTGACACCATCACCAGA

### Relative mRNA quantification

Transcript levels of the 13 mitochondrial protein-coding genes from TT and CT frogs were quantified in a StepOnePlus™ Real-Time PCR System (Life Technologies). For real-time fluorescence quantification, *β-actin* was used as the reference gene (upstream primer: 5′-GATCTGGCATCACACTTTCT-3′, downstream primer: 5′-TGGGTGACACCATCACCAGA-3′) [43, 44]. Quantitative primers and *β-actin* primers are shown in Table 1. Serial dilutions of pooled cDNA from control and low-temperature exposed frogs were exploited to establish standard curves and testing of primers for gene quantification. For *RT-*qPCR, each sample was combined with 10 µL SYBR *Premix Ex Taq* II (2×), 0.4 µL ROX Reference Dye (50×), 0.8 µL each of forward and reverse primers (10 µM), 6 µL ddH_2_O and 2 µL RT reactants (cDNA). Three technical replicates were run for each gene corresponding to each primer. Reactions were carried out under the following conditions: initial denaturation at 95 °C for 30 s, and then 40 cycles of 95 °C for 5 s, 60 °C for 30 s. Relative mRNA quantification was done by calculating the ratio of the starting quantity of the target gene to the starting quantity of the reference gene for each sample.

### Data analysis

Data are expressed as mean expression ± SE for each experimental condition, and four independent biological replicates for each gene were analyzed for the two experimental groups. The relative transcript levels of mRNA in organs at specific stages were analyzed by the 2^−ΔΔCt^ method and standardized to the *β-actin* gene. All data were analyzed using Statistical Program for Social Sciences 21.0 software (SPSS, Inc., Chicago, IL, USA). Gene transcript levels in frog tissues from the control and low-temperature groups were compared using the Student’s *t*-TEST function, and *P* < 0.05 was considered statistically significant [67]. Data are presented graphically using Origin 8.0 software (Origin Lab).

## Data Availability

Raw supplementary data to this article can be found online at https://cloud.zjnu.edu.cn/share/20727a71878dcfc027191f09e4.

## Abbreviations

mt DNA: Mitochondrial DNA
OXPHOS: oxidative phosphorylation
PCGs: Protein-coding genes
PCR: Polymerase chain reaction
*RT*-qPCR: Reverse transcription-quantitative polymerase chain reaction
NCBI: National Center for Biotechnology Information
bp: Base pair
CT frogs: Chinese tiger frogs
TT frogs: Thai tiger frogs
SE: Standard error
*P*: *P*-value
